# Late-Onset Thyroid Hormone Resistance Following Total Thyroidectomy for Papillary Thyroid Cancer

**DOI:** 10.7759/cureus.80673

**Published:** 2025-03-16

**Authors:** Ambika Kapil, Pamella Morello, Maray Rocher, Odalys Frontela, Sahar S Abdelmoneim

**Affiliations:** 1 Osteopathic Medicine, Nova Southeastern University Dr. Kiran C. Patel College of Osteopathic Medicine, Fort Lauderdale, USA; 2 Internal Medicine, Larkin Community Hospital, Hialeah, USA; 3 General Internal Medicine, Larkin Community Hospital, Hialeah, USA; 4 General Internal Medicine/Cardiovascular Medicine, Assiut University Hospital, Assiut, EGY

**Keywords:** delayed presentation, genetic mutations in thyroid hormone signaling, hyperthyroxinemia with normal tsh, thyroid cancer treatment outcomes, thyroid hormone resistance (rth)

## Abstract

Thyroid hormone resistance (RTH) is a rare disorder characterized by impaired cellular responsiveness to thyroid hormones, leading to discordant thyroid function tests and varied clinical manifestations. We present the case of a 43-year-old Cuban-American female patient who presented with dizziness, vertigo, repeated falls, and a severe headache following a minor fall. Additional symptoms included nausea, vomiting, photophobia, cold intolerance, generalized body aches, and fatigue. Her medical history was significant for total thyroidectomy for papillary thyroid cancer, multiple sclerosis (MS), pituitary macroadenoma, Cushing’s disease, and polycystic ovary syndrome (PCOS). Post-thyroidectomy, she remained on high-dose levothyroxine (400-750 mcg daily). However, laboratory tests showed persistently elevated thyroid-stimulating hormone (TSH) levels, hence raising suspicion for RTH.

This case highlights the challenges of diagnosing and managing RTH, a rare endocrine disorder often resulting from mutations in the thyroid hormone receptor-beta (THB) gene. RTH is characterized by inappropriately normal or elevated TSH despite high thyroid hormone levels, reflecting tissue-level resistance. This case underscores the complexities of identifying RTH in the setting of multiple comorbidities and a history of thyroidectomy. It also emphasizes the need for clinician awareness of atypical presentations of RTH, particularly in patients with extensive endocrine and systemic histories.

## Introduction

Thyroid hormone resistance (RTH) is a rare, genetically inherited disorder characterized by impaired tissue sensitivity to thyroid hormones, resulting in elevated levels of thyroid-stimulating hormone (TSH) despite normal or elevated circulating thyroid hormones [1,2]. This condition presents a diagnostic challenge due to its overlap with other thyroid disorders, such as primary hypothyroidism and pituitary adenomas. In RTH, patients often present with elevated free thyroxine (T4) and triiodothyronine (T3) levels alongside normal or inappropriately elevated TSH levels, similar to findings in TSH-secreting pituitary adenomas [3]. However, unlike pituitary adenomas, RTH typically lacks signs of pituitary mass effect (e.g., headaches and visual field defects) and does not suppress TSH in response to exogenous T4 administration [3]. Conversely, in primary hypothyroidism, TSH levels are typically elevated due to reduced negative feedback from low circulating T4 and T3 levels, distinguishing it from both RTH and pituitary adenomas [2]. Recognizing these differences is critical to avoid misdiagnosis and inappropriate treatment. RTH can also coexist with other endocrine abnormalities, complicating the clinical picture. This case report discusses a 43-year-old woman with a history of total thyroidectomy for papillary thyroid cancer, who presented with dizziness, vertigo, and repeated falls, later diagnosed with RTH. This case highlights the importance of recognizing the clinical and laboratory features of RTH in patients with abnormal thyroid function tests, especially those with a complex medical history.

## Case presentation

A 43-year-old female patient presented to the hospital with complaints of dizziness, vertigo, and repeated falls. Her past medical history includes Cushing’s disease and polycystic ovary syndrome (PCOS) with hirsutism diagnosed at an early age, papillary thyroid cancer (treated with total thyroidectomy in 2010), pituitary macroadenoma (treated with transsphenoidal pituitary resection in 2011), legal blindness in the right eye secondary to retinal detachment, and multiple sclerosis (MS) (diagnosed in 2020). Additionally, the patient was diagnosed with RTH seven years ago, by genetic testing and corresponding TSH levels greater than 100 mIU/L, and is being managed by levothyroxine. The patient described the falls as resembling previous episodes of MS relapses, although she did not experience any loss of consciousness or seizure-like symptoms. During her interview, the patient shared that she has smoked about one pack of cigarettes per day for the last 20 years and drinks socially. Additionally, she confirmed a family history of lung cancer on the maternal side, but no history of endocrine disorders. During her hospital stay, she developed severe headaches (intensity 10/10), nausea, vomiting (six episodes in total), and photophobia, which led to further evaluation. Her vital signs showed bradycardia, with a heart rate of 47 bpm, and a normal blood pressure (Table 1). Her physical examination was remarkable for emotional distress, displaying labile mood, irritability, and poor eye contact. She presented signs of hirsutism of the face, delayed relaxation phase of deep tendon reflexes, hypoactive bowel sounds, and a thyroidectomy surgical scar. Her home medications included Synthroid oral 750 mcg daily, sumatriptan 6 mg/0.5 mL subcutaneously for headaches, baclofen 10 mg oral every 12 hours, gabapentin 600 mg oral every 12 hours, Klonopin 1 mg oral every eight hours, and trazodone 100 mg oral every 12 hours.

**Table 1:** Initial hospital admission vital signs

Vital sign	
Blood pressure	106/76 mmHg
Heart rate	47 bpm
Temperature	97.2 deg F
Oxygen saturation	99% on room air
Weight	160 lb
Height	5 ft 4 in
Body mass index (BMI)	27.46 kg/m^2^

**Figure 1:**
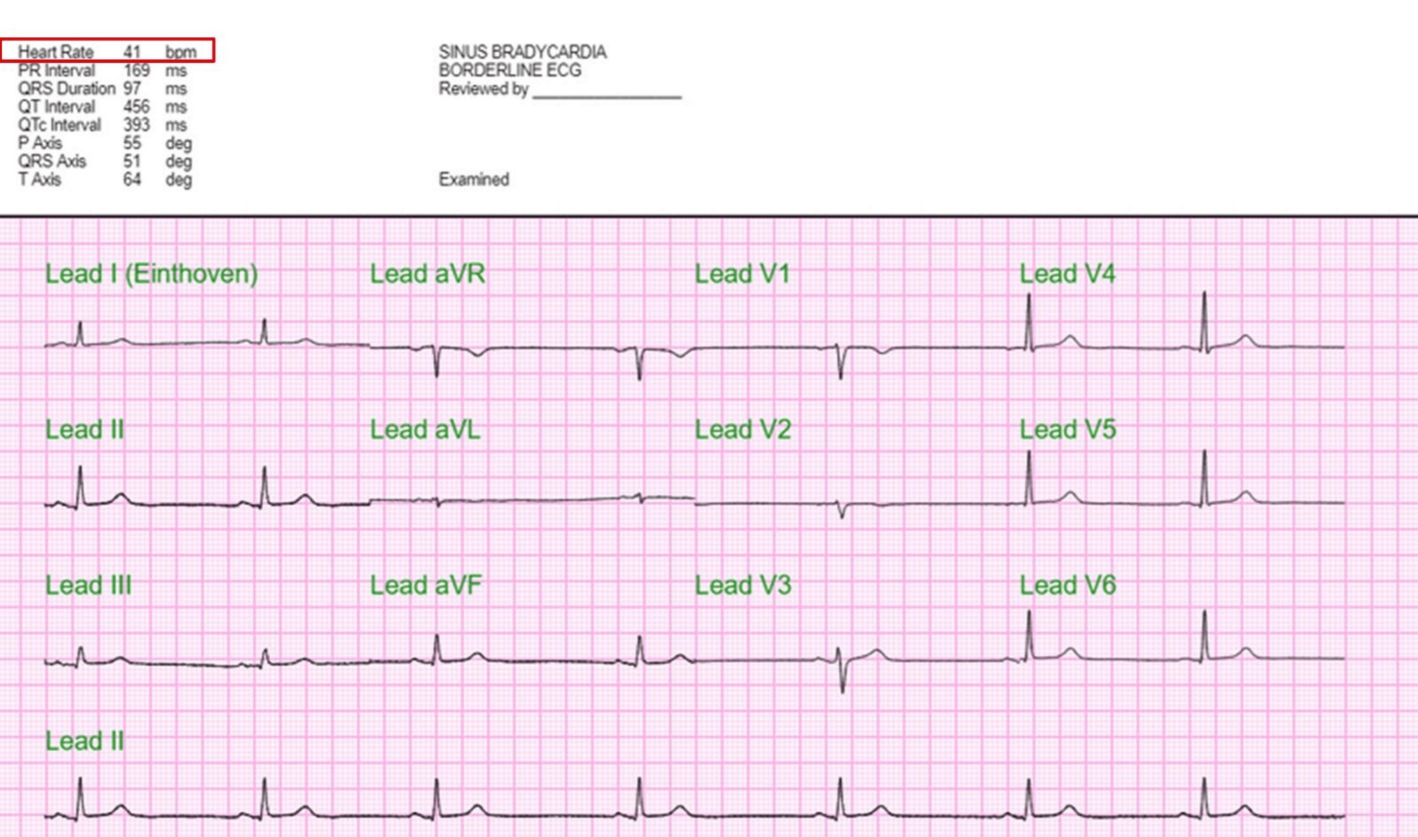
Baseline electrocardiogram (EKG) showing sinus bradycardia with a heart rate of 41 bpm (red box)

**Figure 2:**
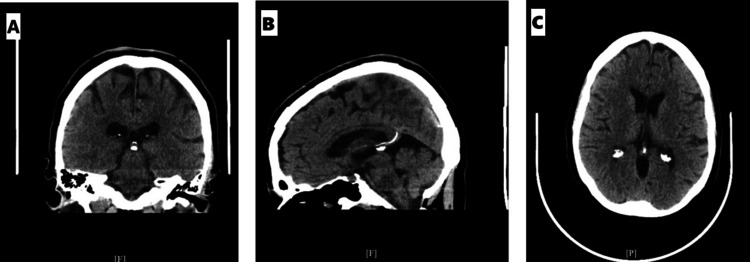
(A-C) Brain CT without intravenous contrast agents. No CT evidence of acute intracranial hemorrhage, midline shift, or mass effect CT: computed tomography

**Figure 3:**
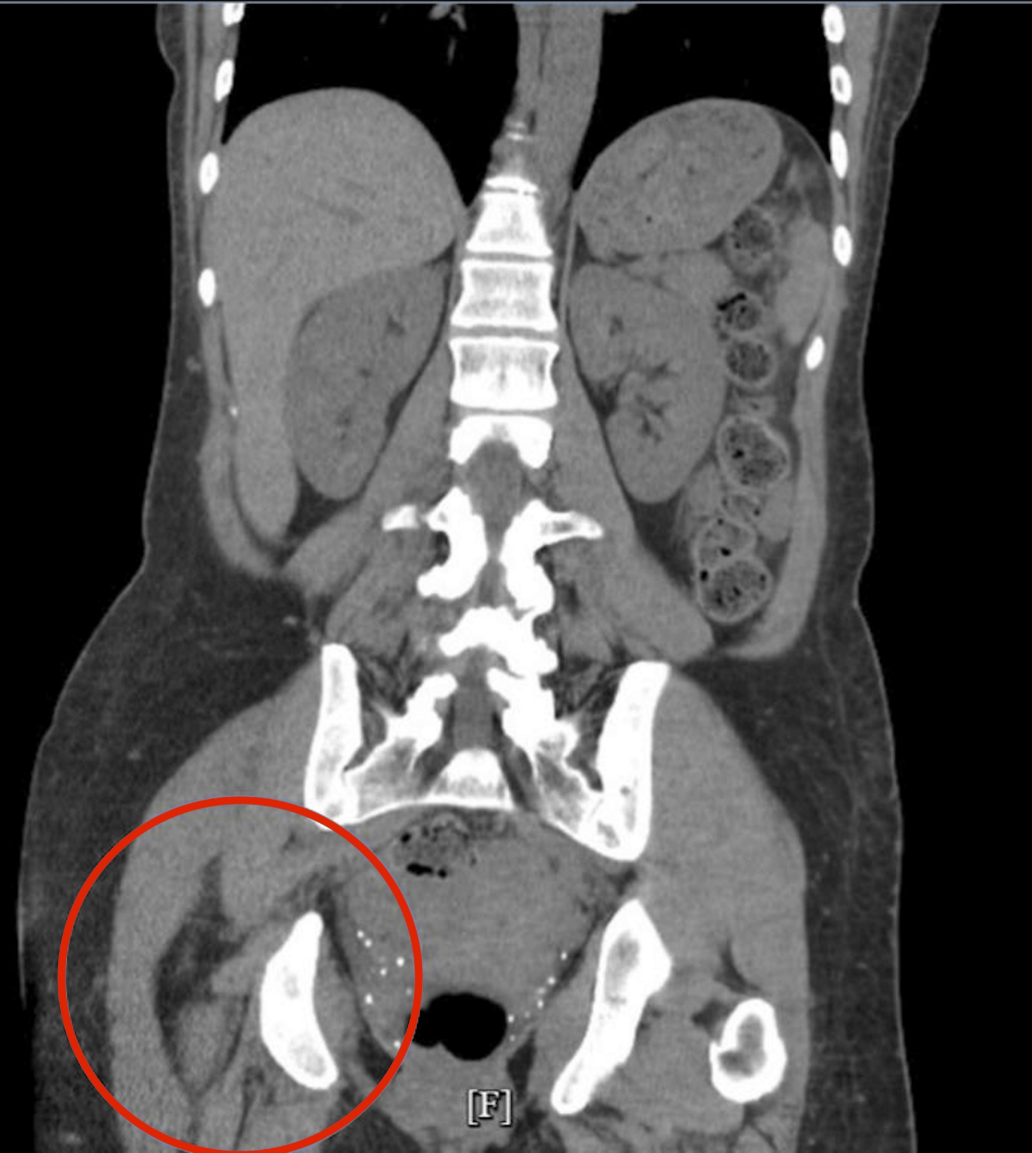
Abdominal CT without contrast. Shows small anterior pelvic soft tissue contusion (red circle), but no other intra-abdominal/adrenal or intrapelvic abnormalities CT: computed tomography

**Figure 4:**
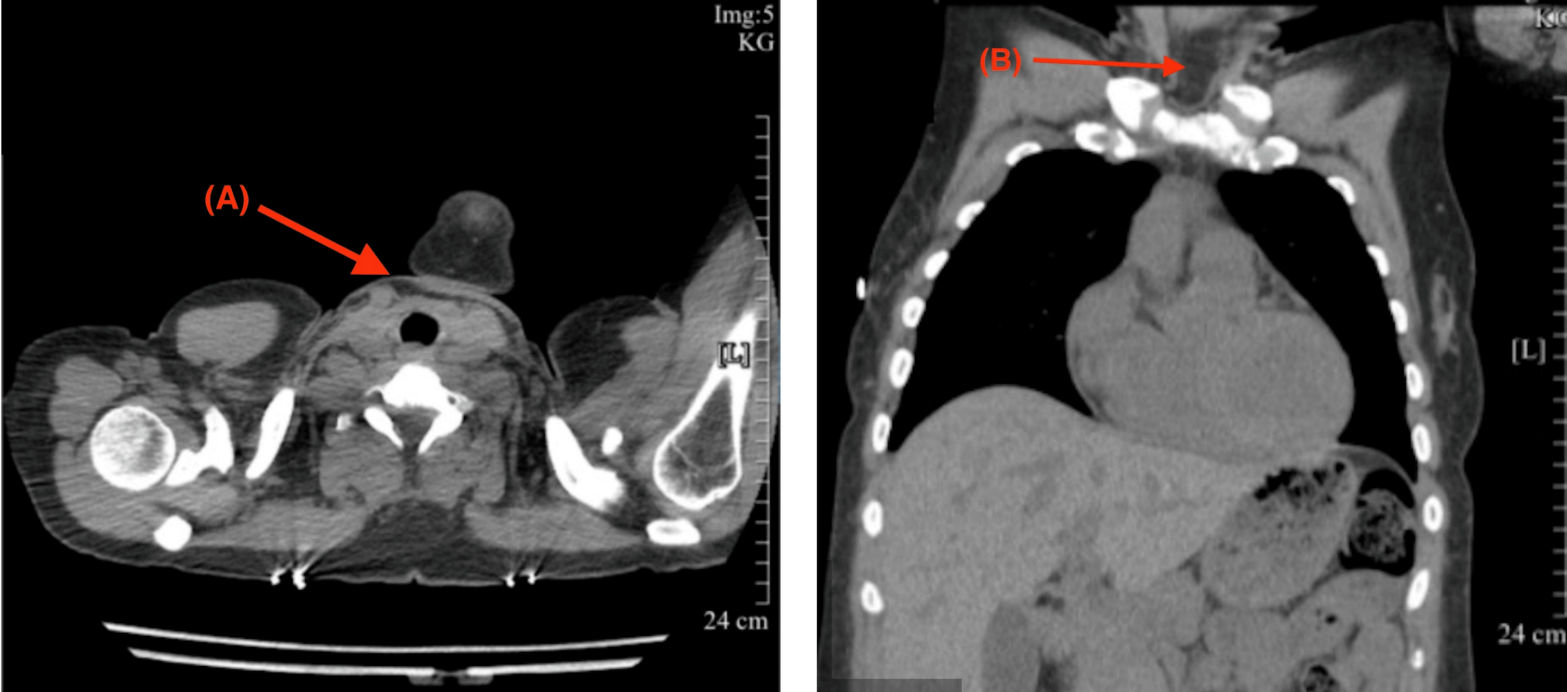
Transverse (A) and anterior-posterior (B) CT chest without intravenous contrast showing no acute cardiopulmonary disease. No definite focal lesions in the thyroid gland region (red arrow) CT: computed tomography

**Figure 5:**
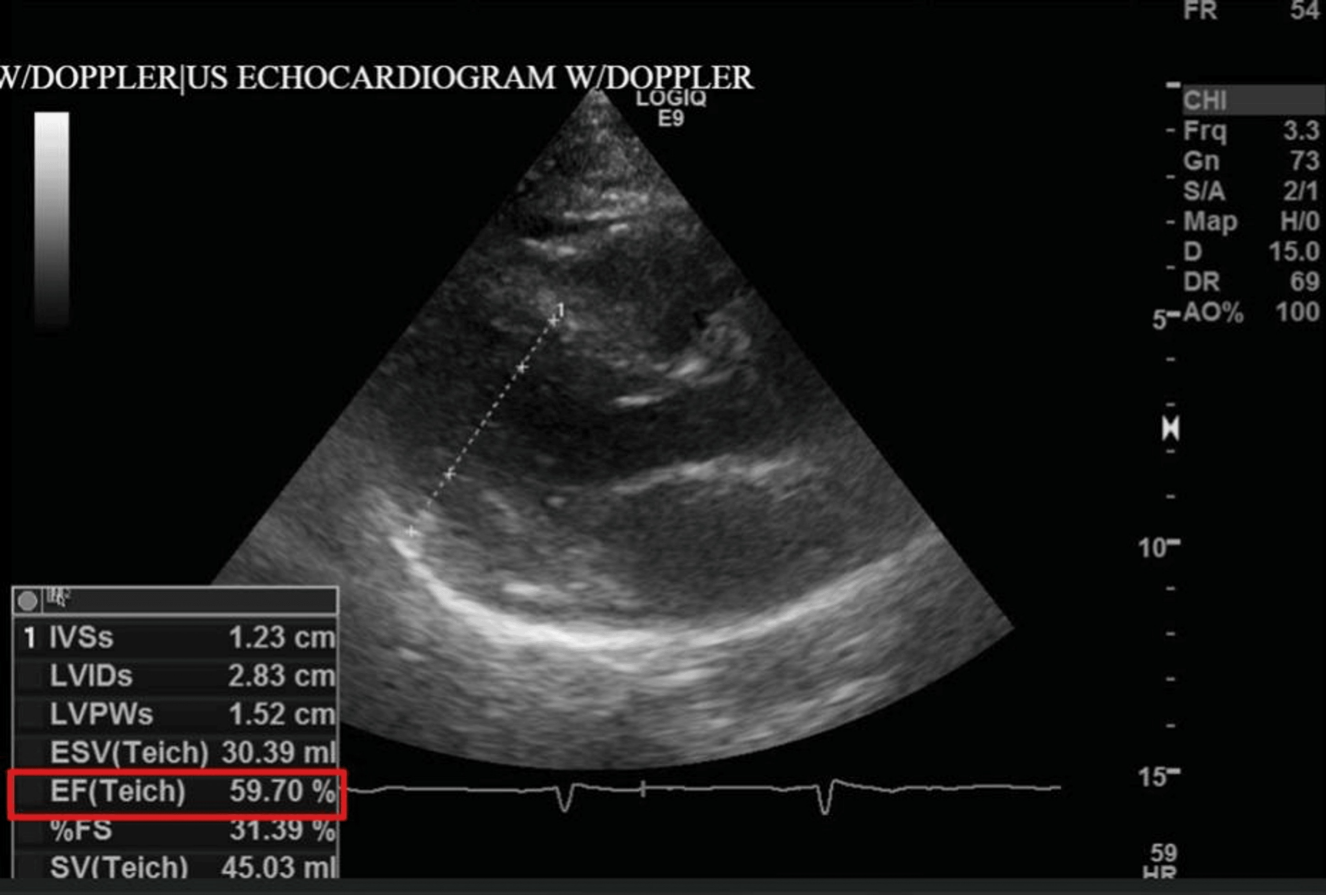
Echocardiogram during hospital admission showed moderate concentric left ventricular hypertrophy and preserved left ventricular ejection fraction (LVEF) (red box)

**Figure 6:**
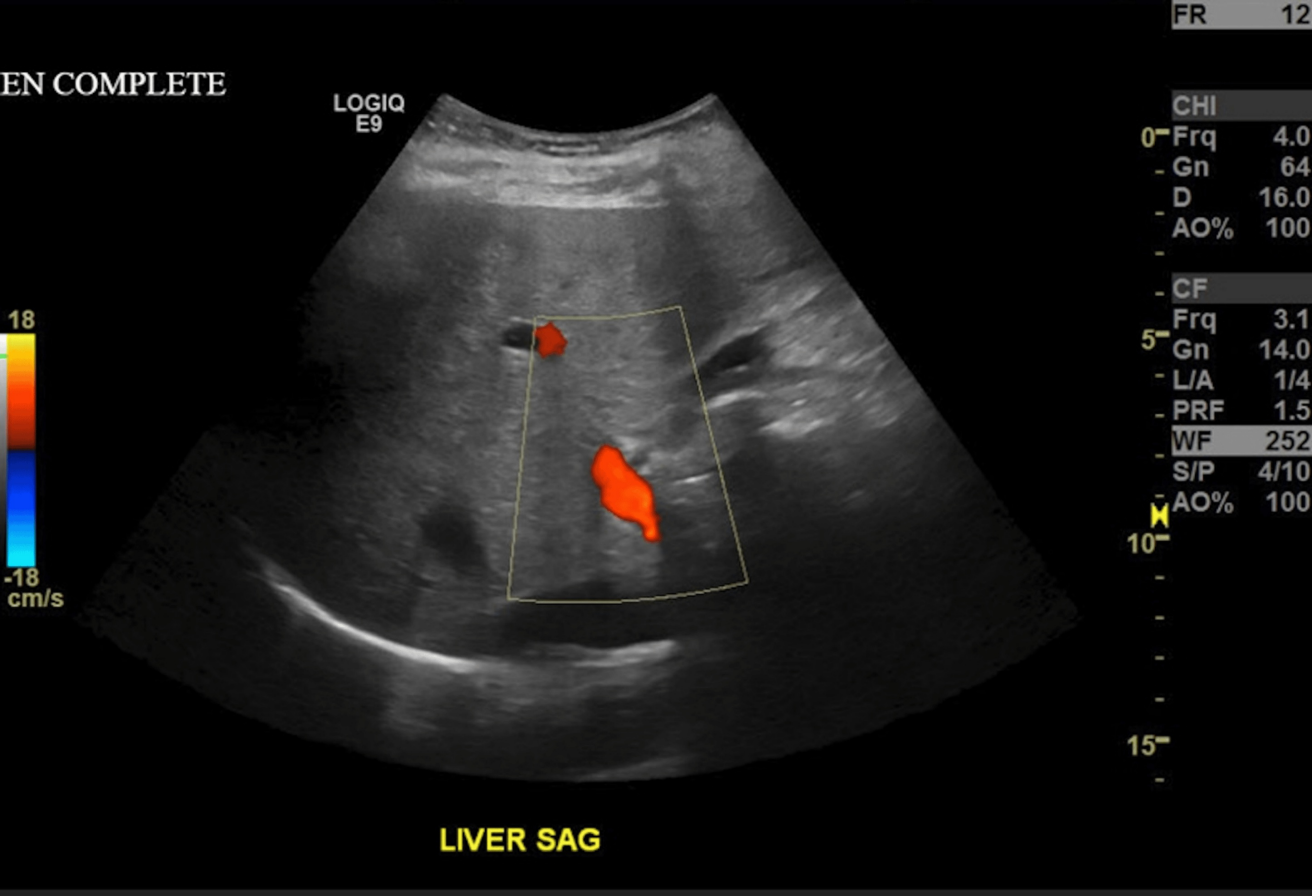
Abdominal ultrasound showed mild hepatomegaly and increased liver echogenicity, which was suspected to represent hepatic steatosis (yellow outline)

Initial laboratory testing revealed elevated TSH levels, consistent with either hypothyroidism or RTH (Table 2). She was started on intravenous (IV) levothyroxine (300 mcg on the day of admission), with daily increases to 800 mcg by hospital day 2. Additionally, due to the patient’s baseline cortisol being below the normal range, an adrenocorticotropic hormone (ACTH) stimulation test and a brain MRI with pituitary protocol were ordered to investigate potential pituitary dysfunction. Throughout her hospitalization, the primary team managed her symptoms with IV fluids, fever/pain control, and nicotine replacement therapy. The electrocardiogram (EKG) showed sinus bradycardia with a heart rate of 41 bpm (Figure 1). Brain computerized tomography (CT) was unremarkable, with no hemorrhage or mass effect (Figure 2). Abdominal CT revealed no acute abnormalities aside from a small anterior pelvic soft tissue contusion (Figure 3). CT chest without contrast showed no acute cardiopulmonary disease and no definite focal lesions in the thyroid gland (Figure 4). Echocardiography demonstrated moderate left ventricular hypertrophy with a preserved left ventricular ejection fraction (LVEF) of 55%-60% (Figure 5). Abdominal ultrasound showed increased liver echogenicity, suggestive of hepatic steatosis or chronic liver disease, and mild hepatomegaly (Figure 6).

**Table 2:** Hospital laboratory values

	Admission	Day 1	Day 2	Reference range
Sodium (Na)	136	135	135	135–145 mmol/L
Potassium (K^+^)	4.7	4.2	4.2	3.5–5.0 mmol/L
Chloride (Cl)	109	110	110	98–107 mmol/L
Bicarbonate (HCO_3_)	22	21	21	22–28 mmol/L
Blood urea nitrogen (BUN)	19	22	14	7–20 mg/dL
Creatinine	0.75	0.90	0.72	0.6–1.2 mg/dL
Glucose	77	72	101	70–99 mg/dL (fasting)
White blood cells	7.6	5.0	7.0	4.5–11.0 × 10³/μL
Neutrophil	67.1	N/A	N/A	30%-75%
Lymphocyte	25.9	N/A	N/A	20%-45%
Monocyte	5.8	N/A	N/A	0%-10%
Eosinophil	0.7	N/A	N/A	0%-6%
Mean corpuscular volume (MCV)	82	N/A	N/A	80-96 fL (female)
Hemoglobin	9.5	9.4	9.3	12.0–15.5 g/dL
Hematocrit	30.8	29.9	30.2	36.0%–44.0%
Platelet	351	321	321	150–400 × 10³/μL
Calcium	8.3	7.9	8.3	8.5–10.5 mg/dL
Total protein	6.5	5.8	6.5	6.0–8.3 g/dL
Albumin	3.3	3.0	3.4	3.5–5.0 g/dL
Aspartate transaminase (AST)	82	49	49	10–40 U/L
Alanine transaminase (ALT)	84	60	53	7–56 U/L
Total bilirubin	0.3	0.2	0.1	0.1–1.2 mg/dL
Direct bilirubin	0.4	N/A	N/A	0.0–0.3 mg/dL
Alkaline phosphatase	50	40	42	44–147 U/L
Urine pH	6.0	N/A	N/A	5-9
Nitrite	Negative	N/A	N/A	Negative
Leukocyte esterase	Negative	N/A	N/A	Negative
Blood	Negative	N/A	N/A	Negative
Ketones	+15	N/A	N/A	Negative
Thyroid-stimulating hormone (TSH)	117.0	>100	N/A	0.4–4.5 µU/mL
Free triiodothyronine (FT3)	0.86	2.95	1.97	2.3–4.1 pg/mL
Free thyroxine (FT4)	0.09	0.82	2.14	0.7–1.7 ng/dL
Thyroid peroxidase (TPO)	N/A	N/A	13	<35.0 IU/mL
Thyroglobulin antibody (TgAb)	N/A	N/A	<1.0	<35.0 IU/mL
Thyroglobulin (Tg)	N/A	N/A	<0.2	3-40 ng/mL
Follicle-stimulating hormone (FSH)	N/A	N/A	4.1	3-9 mIU/mL
Luteinizing hormone (LH)	N/A	N/A	6.0	2-10 mIU/mL
Parathyroid hormone (PTH)	N/A	N/A	18	15-65.0 pg/mL
Vitamin D-1,25 dihydroxy test	N/A	N/A	69.6	18-28.0 pg/dL
Growth hormone (GH)	N/A	N/A	0.2	0.05-8 ng/mL
Insulin-like growth factor 1 (IGF-1)	N/A	N/A	108	88-246 ng/mL
Antidiuretic hormone (ADH)	N/A	N/A	2.8	1-5 pg/mL
Prolactin	49.0	N/A	49.0	0-25 ng/mL
Lactic acid	1.5	N/A	N/A	0.5-1.6 mmol/L
Urine drug screen (UDS)	(+) cocaine	N/A	N/A	Negative
Pregnancy test	Negative	N/A	N/A	Negative
ACTH	N/A	4.1	N/A	9-56 pg/mL
Baseline cortisol	N/A	5	N/A	5-25 μg/dL
30 min cortisol	N/A	17	N/A	≥18 μg/dL
60 min cortisol	N/A	21	N/A	≥18 μg/dL

Further planning for patient treatment was to transition the patient to an oral dose of levothyroxine 300 mcg and to follow up with an endocrinologist. Patient education and counseling were provided about her diagnosis of RTH, with emphasis on the importance of laboratory evaluation of thyroid profile at closer intervals.

## Discussion

RTH is a rare disorder that leads to a mismatch between circulating thyroid hormone levels and tissue sensitivity to these hormones. The diagnosis of RTH is challenging due to its similar presentation to primary hypothyroidism or subclinical hypothyroidism, which also presents with elevated TSH levels [4]. RTH can be suspected when a patient presents with elevated TSH levels in the presence of normal or elevated free T4 and T3 levels. In this case, the patient's abnormal thyroid function tests, along with her clinical presentation of dizziness, fatigue, and cold intolerance, raised suspicion for RTH, despite her history of thyroidectomy.

In the specialty of Endocrinology, interpreting laboratory values requires careful consideration of not just overtly abnormal results but also values that fall within the low-normal or high-normal range. The differential diagnosis for elevated TSH with normal or high thyroid hormone levels includes subclinical hypothyroidism, TSH-producing pituitary adenoma, and RTH syndrome. Suboptimal thyroid hormone therapy is another potential cause, which may be due to factors such as poor medication compliance, drug interactions, or malabsorption syndromes [5]. Low levels of free T3 and T4 made the diagnosis of subclinical hypothyroidism less likely, given that the disease process is characterized by normal levels of free T4. Despite TSH-producing pituitary adenoma presenting similar findings in regard to thyroid hormones as in our patient, the elevated level of prolactin indicates the pituitary as a whole was likely being stimulated. In this scenario, it is hypothesized that despite the intake of levothyroxine, its dosage was either suboptimal, possibly due to medication non-compliance, or the pituitary gland receptors were resistant to T3/T4 hormones, preventing negative feedback from taking place. However, given that this patient had a history of thyroidectomy, RTH was a key consideration. The management approach included adjusting the patient’s levothyroxine dosage to meet the clinical and biochemical needs, although a definitive diagnosis could not be established due to the patient's early discharge.

RTH syndrome is mainly associated with mutations of the thyroid hormone receptor-beta (THB) and less commonly on the thyroid hormone receptor-alpha (THA). These gene mutations are associated with chromosomes 3 and 17, respectively, which can result in mutations of correspondent receptor isoforms THA-1, THA-2, THB-1, and THB-2. Each isoform is expressed more predominantly in specific organs of the body. For instance, THB-1 is more expressed in the brain, liver, and kidneys while THB-2 is expressed in the pituitary gland, hypothalamus, inner ear, and retina. Individuals with THB mutations can also present with phenotypic signs and symptoms, suggesting that other genetic and epigenetic mutations are also associated with the presentation of THB mutation [6,7]. The same goes for THA mutations, in which individuals can present with anemia, constipation, and growth and developmental delays. While our patient did not receive a genetic workup while hospitalized, outpatient follow-up with specialists is appropriate.

Notably, this patient’s presentation differs significantly from previously documented cases of THB mutations in the literature [7]. As summarized in Table 3, most reported cases of RTH feature elevated T4 and T3 levels due to impaired thyroid hormone receptor function. However, our patient is unique in presenting with low free T4 and T3 levels, an unexpected finding that contradicts the typical phenotype of RTH. Additionally, while most RTH patients required levothyroxine doses ranging from 100 to 150 mcg/day post-thyroidectomy, this patient was prescribed an exceptionally high dose of 750 mcg/day (later reduced to 400 mcg/day due to side effects) yet remained clinically unstable and required readmission. This suggests a potentially severe or atypical form of RTH, possibly involving altered thyroid hormone transport, tissue-specific resistance, impaired deiodinase activity, or gastrointestinal malabsorption.

**Table 3:** Review of known cases of RTH patients with differentiated thyroid cancer RTH: thyroid hormone resistance; TSH: thyroid-stimulating hormone; T3: triiodothyronine; T4: thyroxine; PTC: papillary thyroid carcinoma; mPTC: papillary thyroid microcarcinoma; RAI: radioactive iodine ablation; TMNG: toxic multinodular goiter; THB: thyroid hormone receptor-beta; FTC: follicular thyroid carcinoma *Dosage given to the patient during the current hospital admission

Study	Age/gender	RTH mutation	TSH level	Thyroid hormone levels	Cancer type	Management	Levothyroxine dose	Outcome
Vinagre et al., 2014 [7]	19/F	THB mutation	Elevated	Elevated T3/T4	mPTC	Surgery + radioactive iodine (RAI)	100 mcg/d	No recurrence
Ünlütürk et al., 2013 [8]	38/M	THB mutation	Normal	Elevated T4, normal T3	FTC	Surgery + suppressive therapy	125 mcg/d	Stable
	50/F	THB mutation	Inappropriately normal	Elevated free T4	PTC	Surgery only	150 mcg/d	Follow-up ongoing
	42/M	THB mutation	Normal	Elevated T4/T3	FTC	Surgery + RAI	125 mcg/d	No metastasis
	32/F	THB mutation	Normal	Elevated T4, normal T3	PTC	Total thyroidectomy + RAI	100 mcg/day	Disease-free
Fang et al., 2022 [9]	32/F	THB mutation	Normal	Elevated T4/T3	PTC	Total thyroidectomy + RAI	100 mcg/d	Disease-free
	26/F	THB mutation	Non-suppressed	Elevated T4/T3	PTC	Total thyroidectomy	150 mcg/d	Stable
Paragliola et al., 2011 [10]	28/F	THB mutation	Elevated	Elevated T4/T3	PTC	Total thyroidectomy + RAI	125 mcg/d	No recurrence
	35/M	THB mutation	Elevated	Elevated T4/T3	FTC	Total thyroidectomy + RAI	150 mcg/d	No recurrence
	40/M	THB mutation	Elevated	Elevated T4/T3	PTC	Total thyroidectomy + RAI	150 mcg/d	No recurrence
Kim et al., 2010 [11]	30/F	THB mutation	Elevated	Elevated T4/T3	FTC	Total thyroidectomy + RAI	100 mcg/d	Disease-free
Taniyama et al., 2001 [12]	53/F	THB mutation	Normal	Elevated T4/T3	TMNG	Surgery only	125 mcg/d	The patient improved post-surgery
Karakose et al., 2015 [13]	56/F (mother)	THB mutation	Normal	Elevated T4/T3	PTC	Total thyroidectomy	200 md/d	Completely resolved
	33/M (incidental finding in son)	THB mutation	Elevated	Elevated T4/T3	PTC	Total thyroidectomy	100 mg/d	
Current case	43/F	THB mutation	Elevated	Decreased T4/T3	PTC	Thyroidectomy	300 mcg/d*	Status 10 years after thyroidectomy: unstable and readmitted for symptoms

In this case, the patient's symptoms of dizziness, vertigo, and falls may have been exacerbated by her underlying thyroid dysfunction, although it is important to note that these symptoms can also be attributed to other causes, such as MS or vestibular disorders. The patient's psychological symptoms of agitation and poor insight further complicated the clinical picture, highlighting the importance of considering both physical and mental health in managing patients with complex medical histories.

Overall, this case underscores the need for a thorough diagnostic workup in patients with abnormal thyroid function tests, particularly those with complex medical histories or multiple comorbidities. Early recognition of RTH can prevent misdiagnosis and unnecessary interventions, ultimately leading to better patient outcomes.

## Conclusions

This case highlights the complexities involved in diagnosing and managing RTH, especially in patients with a history of thyroidectomy and multiple comorbidities. The patient's presentation of dizziness, vertigo, and falls, combined with elevated TSH and initially low free T4 levels, exemplifies the diagnostic challenges of distinguishing RTH from other thyroid disorders, such as subclinical hypothyroidism or TSH-producing pituitary adenomas. The premature discontinuation of care further underscores the need for patient education and engagement in their treatment plan. Timely recognition of RTH, coupled with tailored therapeutic interventions, is crucial to managing symptoms effectively and preventing long-term complications. This case emphasizes the importance of an interdisciplinary approach and careful monitoring in patients with rare endocrine disorders, ensuring a comprehensive understanding of their unique clinical needs.
